# TCF4 and HuR mediated-METTL14 suppresses dissemination of colorectal cancer via N6-methyladenosine-dependent silencing of ARRDC4

**DOI:** 10.1038/s41419-021-04459-0

**Published:** 2021-12-17

**Authors:** Hao Wang, Wei Wei, Zhong-Yuan Zhang, Yao Liu, Bin Shi, Wen Zhong, Hou-Shun Zhang, Xin Fang, Chun-Lei Sun, Jia-Bei Wang, Lian-Xin Liu

**Affiliations:** 1 Department of Clinical Laboratory, The First Affiliated Hospital of USTC, Division of Life Sciences and Medicine, University of Science and Technology of China, Hefei, China; 2 Department of Hepatobiliary Surgery, Anhui Provincial Clinical Research Center for Hepatobiliary Diseases, Anhui Province Key Laboratory of Hepatopancreatobiliary Surgery, The First Affiliated Hospital of USTC, Division of Life Sciences and Medicine, University of Science and Technology of China, Heifei, China; 3 Department of Radiology, The First Affiliated Hospital of USTC, Division of Life Sciences and Medicine, University of Science and Technology of China, Hefei, China; 4 Department of general surgery, The First Affiliated Hospital of USTC, Division of Life Sciences and Medicine, University of Science and Technology of China, Hefei, China; 5 Department of Pathology, The First Affiliated Hospital of USTC, Division of Life Sciences and Medicine, University of Science and Technology of China, Hefei, China

**Keywords:** Cancer prevention, Post-translational modifications

## Abstract

Metastasis remains the major obstacle to improved survival for colorectal cancer (CRC) patients. Dysregulation of N6-methyladenosine (m6A) is causally associated with the development of metastasis through poorly understood mechanisms. Here, we report that METTL14, a key component of m6A methylation, is functionally related to the inhibition of ARRDC4/ZEB1 signaling and to the consequent suppression of CRC metastasis. We unveil METTL14-mediated m6A modification profile and identify ARRDC4 as a direct downstream target of METTL14. Knockdown of METTL14 significantly enhanced ARRDC4 mRNA stability relying on the “reader” protein YHTDF2 dependent manner. Moreover, we demonstrate that TCF4 can induce METTL14 protein expression, and HuR suppress METTL14 expression by directly binding to its promoter. Clinically, our results show that decreased METTL14 is correlated with poor prognosis and acts as an independent predictor of CRC survival. Collectively, our findings propose that METTL14 functions as a metastasis suppressor, and define a novel signaling axis of TCF4/HuR-METTL14-YHTDF2-ARRDC4-ZEB1 in CRC, which might be potential therapeutic targets for CRC.

## Introduction

Colorectal cancer (CRC) is one of the most common human malignancies with the third leading rate of cancer-related mortality worldwide [1]. Despite great advances in surgical techniques and adjuvant therapy for CRC in recent years, the prognosis is still far from satisfactory due to recurrence and liver metastasis [2]. Therefore, a better understanding of the mechanisms underlying CRC progression will be meaningful for exploring potential therapeutic strategies.

As the most abundant post-transcriptional modification ubiquitously occurring in eukaryotic mRNAs, N6-methyladenosine (m6A) methylation exerts important roles in regulating mRNA stability, pre-mRNA splicing, transport and translation efficiency, as well as RNA-protein interactions [3–5]. As a dynamic and reversible process, m6A modification is triggered by m6A methyltransferases (also called “writers”) and eliminated by demethylases (also called “erasers”). The former mainly includes methyltransferase-like 3/14 (METTL3/14) [6–8], Wilms tumor 1 associated protein (WTAP) and vir-Like m6A methyltransferase associated (KIAA1429) [9–11], and the latter comprises fat-mass and obesity-associated protein (FTO) and alkylation repair homolog protein 5 (ALKBH5) [12–15]. Moreover, m6A modification is dependent on specific RNA-binding proteins (also called “readers”), including YTH domain-containing family protein 1/2/3(YTHDF1/2/3), insulin-like growth factor 2 mRNA binding proteins 1/2/3(IGF2BP1/2/3) and heterogeneous nuclear ribonucleoprotein family (HNRNPA2B1, HNRNPC), etc [16, 17].

M6A modification mainly exerts its biological functions in tissue development, stem cell differentiation, embryonic development, DNA damage, circadian periods, spermatogenesis and so on [18]. Recently, emerging researches have revealed that m6A modification plays an indispensable role in malignancy progression [5, 19]. As a pivotal m6A methyltransferase, METTL14 has recently been reported to be crucial for cancer initiation and progression in hepatocellular carcinoma, bladder cancer and pancreatic cancer by regulating diverse targets [6, 20, 21]. However, the transcriptional regulation of METTL14 and the underlying mechanisms between METTL14, “readers” and “targets” remain largely elusive in CRC.

In this study, we have elucidated the function of METTL14 in suppressing CRC metastasis, and the capability of TCF4 and Hu-Antigen R (HuR) in regulating METTL14 expression. Furthermore, Arrestin domain containing 4 (ARRDC4) has been identified as a downstream m6A target of METTL14, and suppression of METTL14 enhances ARRDC4 mRNA stability via a m6A-YTHDF2 dependent pathway. In conclusion, our research has uncovered that METTL14 is a potential prognostic predictor, as well as a promising therapeutic target for CRC.

## Materials and methods

### Cell culture and reagents

The human CRC cell lines SW480, HCT116, HCT15, HCT8, HT29 were purchased from the American Type Culture Collection (ATCC), SW620, Lovo, RKO and immortalized colon epithelial cell lines NCM460 were obtained from Cell Repository of the Chinese Academy of Sciences (Shanghai). All cells were tested for mycoplasma contamination. HCT116 and HT29 were cultured in McCoys’5A (Hyclone) supplemented with 10% fetal bovine serum (FBS, BI), SW480, SW620, RKO and NCM460 were grown in Dulbecco’s modified Eagle’s medium (DMEM, Gibco) with 10% FBS, and HCT15 and HCT8 were maintained with RPMI-1640 (Hyclone). Cells were treated with Global methylation inhibitors 3-Deazaadenosine (DAA, B6121, APExBIO), the influence on the expression of targets was identified by qRT-PCR and western blot.

### Transfection

Lentiviruses expressing shM14 (shMETTL14) and shCON (empty vector), pcDNA3.1-METTL14 (METTL14-OE) and empty Vector (METTL14-NC) were purchased from Hanbio Biotechnology Co.Ltd (Shanghai). Stable METTL14 knockdown experiment was carried out in SW480 and HCT116 cells, while METTL14 overexpression was conducted in SW620 cells. Transfected cells were selected using puromycin (3 μg/ml) for 7 days or more. Small interfering RNAs (siRNA) targeted HuR, YTHDF2, ARRDC4 and corresponding negative controls (siNC) were synthesized by GenePharma Company (Shanghai) and infected cells utilizing Lipofectamine 2000 reagent (Invitrogen). Relevant applied sequence was presented in Supplementary Table S1.

### Western blotting

Cells or frozen tissue were collected and lysed in pre-cooled RIPA buffer (BB-3201, BestBio) containing protease inhibitor cocktail (HY-K0010, MedChemExpress) and PMSF (HY-B0496, MedChemExpress) on ice for 30 min. Equal Protein samples were added in sodium dodecyl sulfate–polyacrylamide gel electrophoresis (SDS–PAGE) and then were transferred to 0.45μm polyvinylidene difluoride (PVDF) membranes (IPVH00010, Merck Millipore). Soaked in 5% non-fat milk at room temperature for 2 h, the membranes were then incubated with primary antibodies at 4 °C overnight. After washed 3 times with TBST, these membranes were incubated with HRP-conjugated secondary antibodies for 1 h at room temperature. Immunoblots signals were detected using Pierce™ ECL Western Blotting Substrate (32106, Thermo Fisher Scientific). Primary antibodies were listed as: anti-METTL14 (HPA038002, Sigma-Aldrich), anti-HuR (#12582, CST), anti-IGF2BP1 (22803-1-AP, Proteintech), anti-YTHDC1 (14392-1-AP, Proteintech), anti-YTHDF1 (17479-1-AP, Proteintech), anti-YTHDF2 (24744-1-AP, Proteintech), anti-YTHDF3 (25537-1-AP, Proteintech), anti-ARRDC4 (HPA042109, Sigma-Aldrich), anti-LaminB1 (66905-1-lg, Proteintech), anti-PSAT1 (A6707, ABclonal), anti-HSP90AA1 (A13501, ABclonal), anti-ANXA1 (A1118, ABclonal), anti-ZEB1 (21544-1-AP, Proteintech), anti-Snail (13099-1-AP, Proteintech), anti-Slug (#9585, CST), anti-Twist (25465-1-AP, Proteintech), anti-TCF4 (22337-1-AP, Proteintech), anti-GAPDH (60004-1-lg, Proteintech) or β-actin (66009-1-lg, Proteintech) were used as the internal control.

### Quantitative real-time PCR (qRT-PCR) and RNA stability assay

Total RNA of cells was extracted by TRIzol Regent (Invitrogen) according to the previous protocols followed by cDNA synthesis with PrimeScript™ RT Master Mix (RR036A, TAKARA). mRNA expression levels were measured by TB Green® Premix Ex Taq™ II Kit (RR820A, TAKAR) on Applied Biosystems StepOnePlus. Relative RNA amount of each group was calculated using the 2^–ΔΔCt^ method with normalization by GAPDH. For RNA stability assay, cells were seeded in 6-well plates overnight, RNA decay rate was measured using actinomycin D (HY-17559, MedChemExpress) at 5 μg/ml and cells were harvested after incubation at indicated time. The primers used in this study were presented in Supplementary Table S1.

### Transwell assay

Transwell migration and invasion assays were conducted in 24-well plates. 4 × 10^4^ cells in 200 μl serum-starved medium were seeded into the upper chamber (8.0 μm pore size filter, Corning) with or without coated Matrigel (BD, Bioscience), while 600 μl medium containing 10% FBS was placed into the lower chamber. After incubation in 37 °C for 48 h, cells passed through the membrane were immobilized by methyl alcohol and stained with 0.2% crystal violet solution. Subsequently, the penetrated cells were photographed and calculated under Olympus microscope.

### Wound-healing assay

1 × 10^6^ cells in complete medium were cultured in 6-well plates overnight. The confluent monolayers of cells were scratched with a fine pipette tip and photographed. After 48 h, cells migration into the wound was visualized and measured under Olympus microscope and compare it with the size of the initial wound.

### Cell viability assay

Cell viability were explored by Cell Counting Kit-8 (CCK-8, Dojindo Laboratories) according to the manufacturer’s instruction. In brief, 3000 cells were seeded into 96-well plates and incubated at 37 °C overnight. Six multiple wells were applied for assessment of each time point. Subsequently, 10 μl CCK8 reagent was added into different groups of CRC cells and incubated in 37 °C for another 2 h. Absorbance was captured at 450 nm daily to evaluate the proliferation potential of cells. All experiments were conducted in triplicate.

### CRC tissue specimens and clinical data

A total of 72 CRC and corresponding adjacent normal samples were collected after surgical resection in The First Affiliated Hospital of USTC between January 2010 and December 2015. Another cohort including 14 paired CRC and para-cancer were collected in 2019 and underwent protein extraction for western blot analysis of METTL14 and HuR expression. Informed consent had been obtained from each patient before our study and those who received local or systemic treatment were not taken into this study. Our study was approved by the Human Research Ethics Committee of The First Affiliated Hospital of USTC. The clinicopathological characteristics of these enrolled patients were listed in Supplementary Table S2.

### Immunohistochemistry (IHC) analysis

The 4μm-thick serial-sectioned CRC and adjacent normal tissue were subjected to IHC staining according to previous protocol. Briefly, following deparaffinization, rehydration and antigen retrieval, sections were conjugated with primary antibodies at 4 °C overnight. After incubation with secondary and development of Diaminobenzidine (DAB), the staining scores of target proteins were evaluated blindly by two independent pathologists by multiplication of the staining intensity grade (0, 1, 2 or 3 indicated negative, weak, moderate or strong stains, respectively) and proportion of positive stains (0, 1, 2, 3 or 4 implied positive areas of 0–5%, 6–25%, 26–50%, 51–75% or 76–100%, respectively).

### MeRIP-sequencing

MeRIP-sequencing projects and subsequent data analysis were supported by Genesky Biotechnologies Inc. (Shanghai, China). Total RNA was isolated from SW480 cells transfected with shMETTL14-2 (shM14-2) or shControl (shCON), followed by poly (A) + RNA purification and fragmentation using NEBNext Poly (A) mRNA Magnetic Isolation Kit (New England Biolabs, UK). Concentration of RNA was detected on Nanodrop 2000 (Thermo Fisher Scientific, USA) and the integrality was guaranteed by Agilent 2100 Bioanalyzer (Agilent Technologies, USA). Dynabeads Protein A (Thermo Fisher Scientific, USA) was mixed with rabbit anti-m6A antibody (Synaptic system, Germany) at 4 °C for 2 h in advance, then fragmented mRNA was incubated with the mixture for another 2 h to precipitate m6A-enriched RNAs. Qualified samples underwent Library Pooling and Sequencing using Illumina HiSeq 2500 machines. Following quality filter, the raw sequence data was mapped to human genome GRCh37/hg19 utilizing the HISAT2 software (v2.0.5) and the results were subjected to analyzed bioinformatically and statistically. The peak calling data and RNA-sequencing data were described in Supplementary Materials.

### m^6^A dot blot assay

Total RNA was extracted as described above and the concentration was determined by NanoDrop one (Thermo Fisher scientific, USA). Samples continuously diluted (200 ng/μl,100 ng/μl and 50 ng/μl) were denatured at 95 °C for 5 min and loaded on the Amersham Hybond-N + membrane (GE Healthcare, USA). Afterwards the membrane was crosslinked by UV light for 10 min followed by blocking with 5% BSA, special m6A antibody (ab, Abcam) was hatched with the membrane at 4 °C overnight. 0.02% Methylene blue staining of the membrane was regarded as RNA loading control. Following incubation with HRP-conjugated anti-rabbit immunoglobulin G for 1 h at room temperature, the signal of dot blot was detected referencing western blot.

### RNA immunoprecipitation (RIP)

RIP experiments were conducted in SW480 and HCT116 cells with the Imprint® RNA Immunoprecipitation Kit (RIP-12RXN, Sigma-Aldrich) following the manufacturer’s illustrations. Briefly, 4 μg specific antibodies against rabbit immunoglobulin G (I5006, Sigma-Aldrich), METTL14, YTHDF2 or m6A were incubated with cell lysates overnight at 4 °C with rotation, then 20 μl washed magnetic beads were added to each reaction and incubated at 4 °C for 2 h. After washed 6 times, interested RNAs in the immunoprecipitation complex were purified for further analysis by qRT-PCR. Relative enrichment of RNA was normalized to the input.

### RNA pull-down assay

RNA pulldown assay was employed to identified the endogenic binding of RNAs and proteins in this study. 3 μg Biotin-labeled probe was incubated with 2 mg cell lysates containing cocktail and Ribonuclease Inhibitor at 4 °C overnight on a rotator, then 20 μl pre-cleared streptavidin magnetic beads (88816, ThermoFisher Scientific) were placed into the lysate for precipitation of RNA-protein complex. The beads were washed with lysis buffer for 4 times and boiled with protein loading buffer for 10 min. Immunoblotting assay was used to determine the efficiency of co-precipitated proteins. The biotin probe sequence: CAUAGAUUGGAAUAGCUUCUC, negative control: GAGAAGCUAUUCCAAUCUAUG.

### Chromatin immunoprecipitation (ChIP) assay

ChIP assay was carried out in SW480 and HCT15 cells with SimpleChIP® Enzymatic Chromatin IP Kit (#9003, CST) following the manufacturer’s instruction. 1 × 10^7^ cells crosslinked by 37% formaldehyde (final concentration was 1%) were lysed in SDS lysis buffer and sonicated to break into DNA fragments with 200–900 bp optimal length. Then the lysate was centrifuged and the supernatant was diluted with ChIP buffer for incubation with anti-HuR antibody or isotype control IgG (#2729, CST) at 4 °C overnight. Next, 30 μl ChIP-grade Protein G magnetic beads were added to the DNA-protein complexes rotating for another 2 h. After washed and eluted, the immunoprecipitates were de-crosslinked at 65 °C for 3 h or overnight. Subsequently, DNA was purified and subjected to qRT-PCR analysis.

### Animal experiments

Subcutaneous xenograft model was performed using 5-weeks old male BALB/c athymic nude mice. These mice were randomly divided into experimental group and control group. Equal amount of HCT116 cells (2 × 10^6^) stably expression of relevant plasmids was injected into the right flank of mice, tumor bulks was monitored once a week after injection and volumes were counted as 0.5 × a^2^ × b (a and b respectively indicated short and long diameter of tumor). Four weeks later, mice were sacrificed and the weight of deprived tumors from each group were obtained. To explore the metastasis abilities of transfected HCT116 cells in vivo, 1 × 10^6^ cells in 100 μl PBS were injected into the tail of mice and the metastatic foci on liver of mice were analyzed after six weeks. The liver tissues were immobilized by formaldehyde, embedded in paraffin and subjected to HE staining. Our study was approved by the Human Research Ethics Committee of The First Affiliated Hospital of USTC.

### Statistical analysis

All of the statistical analyses in this study were operated with the SPSS 19.0 (SPSS, Inc., USA) and GraphPad Prism 5.0 (GraphPad, Inc., USA). Experiments were repeated at least three times. Data were showed as the means ± SD using a two-tailed Student’s t test after homogeneity of variances test. One-way ANOVA was used to compare the differences among the three groups. The correlation between METTL14 protein levels of tissue and clinicopathologic characteristics was identified by Chi-square test. Independent prognostic factors of multiple multivariate analysis were explored with the Cox regression model. Overall survival curve was depicted with Kaplan–Meier analysis and the difference were detected via log-rank test. A *P* value < 0.05 was considered as statistically significant.

## Results

### Decreased METTL14 expression correlates with poor prognosis of patients with CRC

**Fig. 1:**
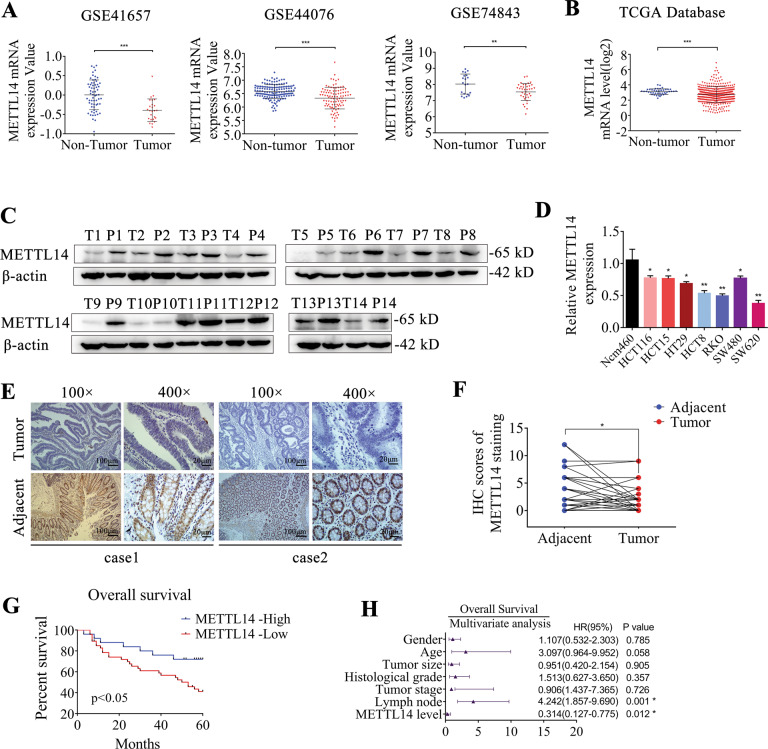
METTL14 is downregulated in CRC and correlated with poor survival. **A** METTL14 expression in the GSE41657, GSE44076 and GSE74843 CRC cohorts. **B** METTL14 expression in the TCGA CRC cohort. **C** METTL14 protein expression was verified by western blot in 14 paired CRC tissue (T) and para-cancerous tissue (P). **D** qRT-PCR analysis of METTL14 mRNA expression in CRC cell lines and intestinal epithelium cells Ncm460. **E** Representative images of immunohistochemistry (IHC) staining in 72 pairs of CRC and adjacent tissue (magnification,100× and 400×). **F** IHC scores of METTL14 staining in CRC and adjacent tissue. **G** Kaplan–Meier survival curves of OS in 72 CRC patients based on METTL14 staining. **H** Multivariate analysis of CRC patients was shown based on COX regression model, several factors correlated with clinical outcomes of CRC were introduced to the model. *P < 0.05, **P < 0.01, ***P < 0.001.

We first analyzed the gene expression pattern of METTL14 in CRC using GEO database (GSE41657, GSE44076 and GSE74843). As shown in Fig. 1A, METTL14 was remarkably downregulated in CRC tissue compared with normal tissue. Upon analyzing the TCGA database, the expression of METTL14 was also found to be decreased in CRC samples (Fig. 1B). The METTL14 expression was gradually decreased during cancer progression (Supplementary Fig. S1A). Further validation showed that METTL14 protein level was downregulated in representative CRC patient tissue compared with adjacent para-cancer tissue (Fig. 1C). The METTL14 mRNA expression in CRC cell lines was also downregulated compared with the normal colonic epithelial cells (Fig. 1D). Similarly, the METTL14 protein expression in a majority of CRC cell lines was lower than in the normal colonic epithelial cells (Supplementary Fig. S1B, C). In addition, the results from IHC staining of 72 pairs of CRC samples and adjacent para-cancer tissue further validated that METTL14 expression was notably reduced in CRC tissue (Fig. 1E, F). Moreover, decreased METTL14 protein was significantly correlated to tumor stage (Supplementary Table S2). Kaplan–Meier survival analysis found that CRC patients with decreased METTL14 expression showed a poor overall survival (Fig. 1G). Survival analysis from TCGA database validated lower METTL14 expression level was associated with worse overall survival (Supplementary Fig. S1D). Importantly, the multivariate Cox regression analysis identified METTL14 expression as an independent predictor of survival for CRC patient (Fig. 1H). Taken together, it can be concluded that METTL14 expression was decreased in CRC and might be involved in CRC progression.

### Suppression of METTL14 promotes CRC metastasis

**Fig. 2:**
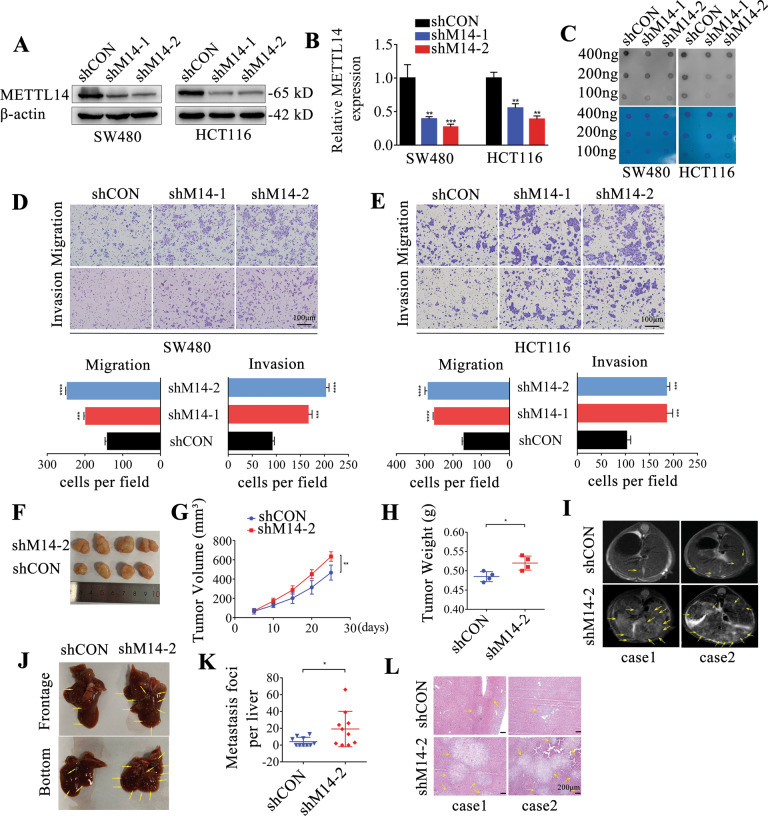
METTL14 depletion promotes CRC metastatic in vitro and in vivo. The knockdown efficiency of METTL14 was confirmed by western blot (**A**) and qRT-PCR (**B**) after lentivirus infection in SW480 and HCT116 cells. **C** Total RNA isolated from METTL14-knockdown CRC cells were subjected to m6A dot blot assay. Methylene blue staining acted as a loading control. Representative images and quantification of transwell assay of METTL14-konckdown SW480 (**D**) and HCT116 cells (**E**). scale bars, 100 μm. **F**–**H** Xenografts derived from HCT116-shM14 or shCON cells (*n* = 4). Tumor volume was recorded at indicated time to establish a growth curve (**G**) and tumors weight (**H**) were measured after mice sacrificed. **I**–**L** Analysis of metastatic liver nodules after the tail vein injection of METTL14-knockdown or control HCT116 cells (*n* = 10 mice per group), evident metastatic lesions were indicated with yellow arrows. **I** Different groups of mice were subjected to magnetic resonance imaging (MRI) scans and the cross section drawing of mice were exhibited. **L** Hematoxylin-eosin staining (HE) of metastatic liver tissue. *P < 0.05, **P < 0.01, ***P < 0.001, ****P < 0.0001.

To unravel the role of METTL14 in CRC progression, the expression of METTL14 was knocked down using two shRNAs (shM14-1, shM14-2) in SW480 and HCT116 cells (Fig. 2A, B). Meanwhile, the expression of METTL14 was elevated using pcDNA3.1-METTL14 vector in SW620 cells (Supplementary Fig. S2A). M6A dot blot assay showed that knockdown of METTL14 reduced m6A modification in CRC cells (Fig. 2C). As illustrated by the transwell assay and wound-healing assay, inhibition of METTL14 significantly promoted migration, invasion and metastasis of SW480 and HCT116 cells (Fig. 2D, E and Supplementary Fig. S2B). In contrast, overexpression of METTL14 attenuated migration, invasion and metastasis of SW620 cells (Supplementary Fig. S2C, 2D). However, METTL14 had no obviously effects on proliferation of CRC cells in vitro (Supplementary Fig. S2E). The function of METTL14 was further evaluated in the METTL14-knockdown CRC xenograft, which indicated that suppression of METTL14 elevated tumor growth, as reflected by tumor size, volume and weight (Fig. 2F–H and Supplementary Fig. S2F). Moreover, we assessed the effect of METTL14 on CRC metastasis in vivo, and the results demonstrated that suppression of METTL14 significantly induced CRC liver metastasis in CRC xenograft derived from HCT116 cells with low METTL14 expression, as indicated by magnetic resonance imaging (MRI) scans (Fig. 2I) and the number of liver metastatic lesions (Fig. 2J–L).

### METTL14 exerts transcriptomic and m6A epigenetic impact on CRC cells

**Fig. 3:**
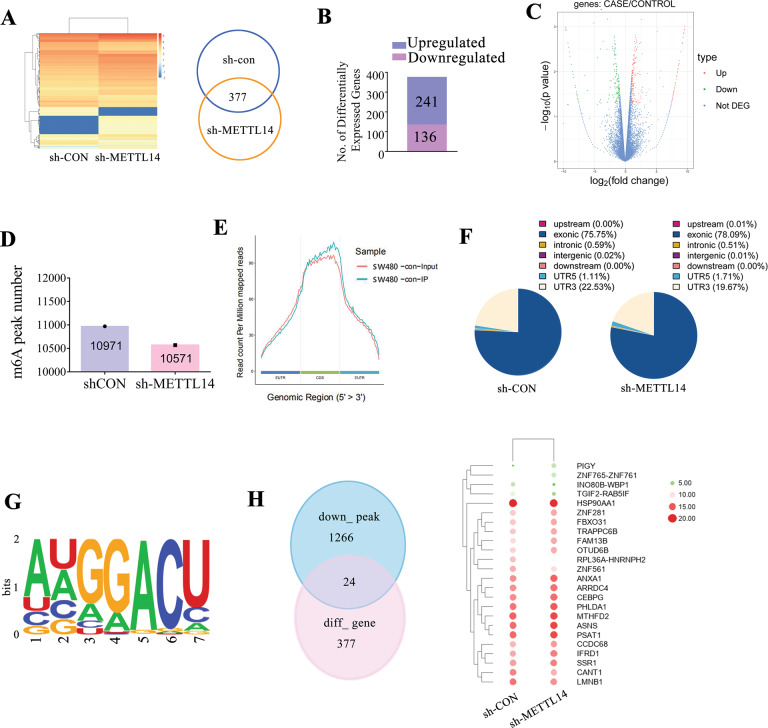
MeRIP-sequencing and RNA-sequencing combination interrogate the candidate target of METTL14. **A**, **B** RNA-sequencing determined differentially expressed genes between shM14 and shCON SW480 cells. **C** The statistically upregulated (red) and downregulated (green) genes were exhibited via volcano plot. **D** m6A peak number were detected in METTL14-knockdown group and control group. **E** Metagene profiles of m6A enrichment across mRNA transcriptome in SW480 cells. **F** Distribution and percentage of the differential peak in the genome. **G** The m6A consensus sequence motif was identified with m6A-sequecing data. **H** Heatmap showed 24 candidate genes came from the intersection of RNA-sequencing and MeRIP-sequencing.

To explore the molecular mechanisms underlying METTL14-mediated metastasis of CRC, RNA-sequencing and MeRIP-sequencing were performed in CRC cells with stable low expression of METTL14 and control cells. RNA-sequencing data showed that 377 transcripts were dysregulated due to METTL14 inhibition (Fig. 3A). Among them, 241 genes were upregulated, while 136 genes were downregulated (Fig. 3B, C and Supplementary Fig. S3). In addition, MeRIP-sequencing identified 10971 and 10571 m6A peaks in control and METTL14-deficient cells, respectively (Fig. 3D). These m6A peaks enriched close to the stop codons and mainly located in exon region (Fig. 3E, F). Moreover, suppression of METTL14 decreased m6A modification in UTR3’ from 22.53% to 19.67% (Fig. 3F). The identified m6A consensus motif (GGAC) implied the successful enrichment of m6A modified mRNA (Fig. 3G). Intriguingly, 24 genes were overlapped in 1266 diminished m6A peaks from MeRIP-sequencing data and 377 dysregulated transcripts from RNA-sequencing (Fig. 3H). Overall, these results indicated that METTL14 inhibition exerted transcriptomic and m6A epigenetic impact on CRC cells.

### ARRDC4 is a m6A target of METTL14

**Fig. 4:**
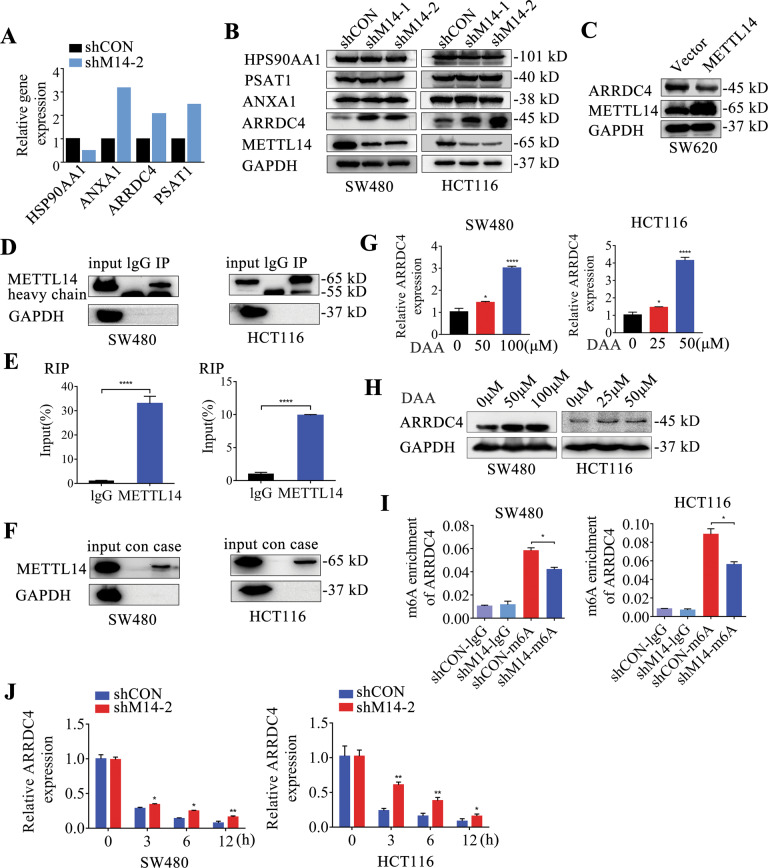
ARRDC4 is identified as the direct target of METTL14. **A** Four differentially expressed candidate genes HSP90AA1, ANXA1, ARRDC4 and PSAT1 were showed according to the gene FPKM in RNA-sequencing data. **B** The protein expression of HSP90AA1, ANXA1, ARRDC4 and PSAT1 in METTL14-deficient SW480 and HCT116 cells were detected by western blot. **C** The protein expression of ARRDC4 in METTL14-overexpressed SW620 cells were detected using western blot. **D**, **E** RIP assay was performed to reveal the relative enrichment of ARRDC4 mRNA associated with METTL14 protein. IgG antibody was used as negative control. IP efficient of METTL14 protein was verified via western blot. **F** Immunoblotting of METTL14 after RNA pull down assay with biotinylated-ARRDC4 in SW480 and HCT116 cells. SW480 and HCT116 cells were treated with different concentration of 3-deazaadenosine (DAA) and ARRDC4 mRNA and protein expression were detected by qRT-PCR (**G**) and western blot (**H**). **I** Relative m6A enrichment of ARRDC4 mRNA was analyzed and normalized to input by using MeRIP-qPCR. Silence of METTL14 decreased m6A abundance on ARRDC4 compared with control group. **J** Stability of ARRDC4 mRNA was detected in METTL14-konckdown and control cells via qRT-PCR at the indicated time after actinomycin D (5 μg/ml) treatment. *P < 0.05, **P < 0.01, ****P < 0.0001.

Among the 24 potential genes, HSP90AA1, ANXA1, ARRDC4 and PSAT1 were involved with cancer progression [22–25]. Knockdown of METTL14 decreased HSP90AA1 mRNA expression and increased ANXA1, ARRDC4 and PSAT1 mRNA expression (Fig. 4A). At the protein level, knockdown of METTL14 enhanced ARRDC4 expression, but had no effect on the HSP90AA1, ANXA1 and PSAT1 (Fig. 4B), and METTL14 overexpression reduced ARRDC4 expression (Fig. 4C). Thus, ARRDC4 was selected for further study as a presumable target of METTL14 for further investigation. Upon separation of nuclear and cytoplasmic protein, ARRDC4 was determined to be located in cytoplasm, and the suppression of METTL14 upregulated cytoplasmic ARRDC4 expression in CRC cells (Supplementary Fig. S4). As revealed by both the RIP assay and the further RNA pull-down assay, METTL14 was capable of binding to ARRDC4 mRNA in CRC cell lines (Fig. 4D, E, F). Consistent with knockdown of METTL14, the treatment of CRC cell lines with 3-deazaadenosine (DAA), a global methylation inhibitor, substantially increased the ARRDC4 mRNA and protein expression in CRC cells (Fig. 4G, H). Furthermore, MeRIP-qPCR assay was performed to detect the m6A abundance in ARRDC4 mRNA, and the results showed that knockdown of METTL14 remarkably diminished ARRDC4 m6A enrichment in CRC cells (Fig. 4I). Additionally, the effect of METTL14 deficiency on decay rate of ARRDC4 mRNA was assessed. As shown in Fig. 4J, METTL14 inhibition markedly elevated the stability of ARRDC4 mRNA and delayed its degradation rate in CRC cells. Taken together, our findings suggested that ARRDC4 is a downstream target of METTL14, and the expression and gene stability of ARRDC4 was modulated by METTL14 in an m6A-dependent manner.

### METTL14 loss stabilizes ARRDC4 mRNA in an m6A-YTHDF2-dependent manner

**Fig. 5:**
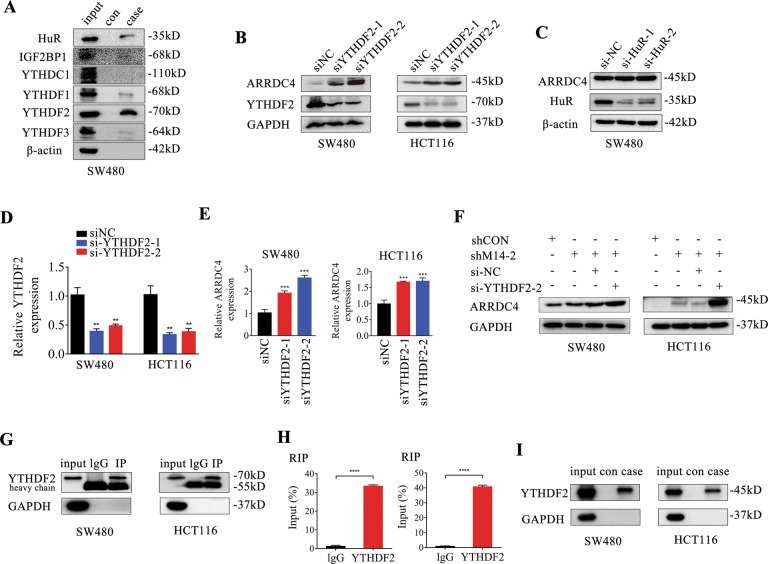
METTL14-modified ARRDC4 is specially recognized by YTHDF2. **A** Immunoblotting of HuR, IGF2BP1, YTHDC1, YTHDF1, YTHDF2, YTHDF3 after RNA pull down assay with cell lysate (input), biotinylated-ARRDC4 (case), and beads only (con) in SW480 cells. **B** Immunoblotting of YTHDF2 and ARRDC4 in SW480 and HCT116 cells with YTHDF2 knockdown. **C** The expression of ARRDC4 and HuR were detected by western blotting in SW480 cells transfected with HuR siRNAs. **D** qRT-PCR analysis confirmed the knockdown efficiency of YTHDF2. **E** YTHDF2 loss enhanced ARRDC4 mRNA expression in SW480 and HCT116 cells. **F** ARRDC4 protein expression was further promoted when downregulated YTHDF2 in METTL14-deficient SW480 and HCT116 cells. **G**, **H** RIP-qPCR displayed the relative enrichment of ARRDC4 mRNA in each group precipitated with lgG or YTHDF2 antibody with the normalization to input. IP efficiency of YTHDF2 was validated using western blot. GAPDH was used as protein control. **I** RNA pulldown assay was conducted to further show that YTHDF2 could specially recognized ARRDC4. GAPDH was used as a loading control. **P < 0.01, ***P < 0.001, ****P < 0.0001.

Mechanisms responsible for m6A regulated mRNA stability were further investigated. It has been revealed that m6A modification may regulate the mRNA stability via readers including YTHDF1-3, YTHDC1, IGF2BP1, and HuR [26, 27]. Results showed that YTHDF2 and HuR, while not YTHDC1, IGF2BP1, or YTHDF1/3, can significantly bind with ARRDC4 mRNA in SW480 cells (Fig. 5A). Furthermore, knockdown of YTHDF2, but not HuR, significantly upregulated ARRDC4 protein expression in CRC cells (Fig. 5B, C). YTHDF2 loss also increased ARRDC4 mRNA expression (Fig. 5D, E). In addition, METTL14 inhibition increased ARRDC4 protein expression and YTHDF2 loss enlarged this phenomenon (Fig. 5F). Moreover, the results of RIP assay revealed that YTHDF2 could directly combine to ARRDC4 mRNA (Fig. 5G, H). Consistently, RNA pulldown assay further confirmed the close interaction between YTHDF2 protein and ARRDC4 mRNA (Fig. 5I). Collectively, our data suggested that the methylated ARRDC4 mRNA were directly identified by the m6A “reader” YTHDF2, which promoted the transcript degradation and naturally decreased its expression via an m6A-YTHDF2-dependent mechanism.

### ARRDC4 weakens the effects of METTL14 in CRC cells

**Fig. 6:**
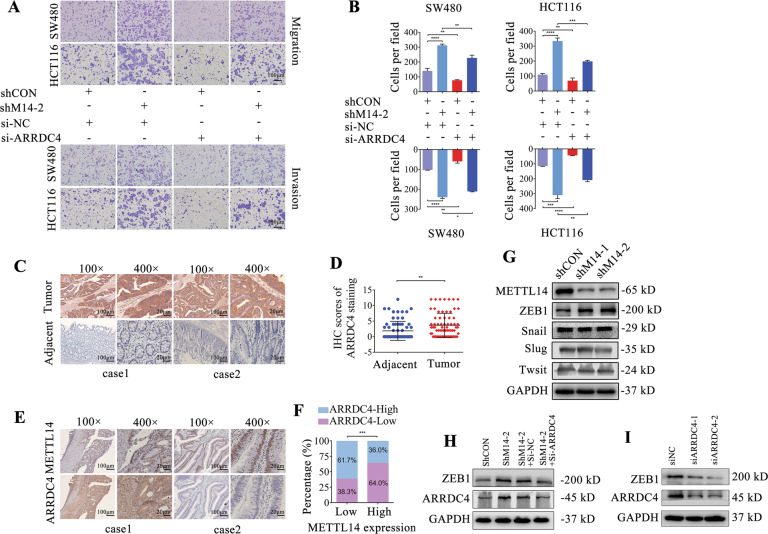
METTL14-mediated ARRDC4 expression is responsible for the metastasis of CRC. **A**, **B** Transwell assay was conducted to measure migration and invasion of CRC cells. Representative images (**A**) and quantification results (**B**) of METTL14-knockdown cells transfected with ARRDC4 siRNAs or their corresponding controls. Scale bars,100 µm. Representative images (**C**) and scores (**D**) of IHC staining for ARRDC4 protein in tumor and adjacent normal tissue (magnification,100× and 400×). **E**, **F** ARRDC4 and METTL14 protein levels were detected by IHC analysis. **G** The expression levels of ZEB1, Snail, Slug, Twist and METTL14 in METTL14-knockdowned SW480 cells were detected by western blotting. **H** The expression levels of ZEB1 and ARRDC4 in METTL14-knockdown SW480 cells transfected with ARRDC4 siRNAs or their corresponding controls were detected by western blot. **I** The expression levels of ZEB1 and ARRDC4 in SW480 cells transfected with ARRDC4 siRNAs or their corresponding controls were detected by western blot. *P < 0.05, **P < 0.01, ***P < 0.001, ****P < 0.0001.

To investigate the oncogenic role of ARRDC4 in CRC, two siRNAs were used to repress the mRNA and protein expression of ARRDC4 in SW480 and HCT116 cells (Supplementary Fig. S5A, B). According to the transwell assay, ARRDC4 loss dramatically weakened knockdown of METTL14-induced migration, invasion and metastasis of SW480 and HCT116 cells (Fig. 6A, B). In addition, the results from IHC staining indicated that ARRDC4 expression was notably increased in CRC compared with adjacent para-cancer tissue (Fig. 6C, D). Moreover, the expression of METTL14 and ARRDC4 exhibited a remarkable negative correlation in CRC samples (Fig. 6E, F). Epithelial-to-mesenchymal (EMT) is an important biological process tightly associated with metastasis capacity of cancer cells. In present study, the EMT related transcriptional factors Snail, Slug, Twist and ZEB1 were detected in METTL14 knockdown cells. The WB results showed that depletion of METTL14 elevated the expression of ZEB1, but not the others (Fig. 6G), while silencing of ARRDC4 could reverse this promotion caused by METTL14 knockdown (Fig. 6H). Suppression of ARRDC4 alone also decreased ZEB1 expression (Fig. 6I). The above results suggested that ARRDC4 played an important role in metastasis mediated by METTL14 deficiency in CRC cells.

### HuR downregulates METTL14 expression by directly binding to its promoter

**Fig. 7:**
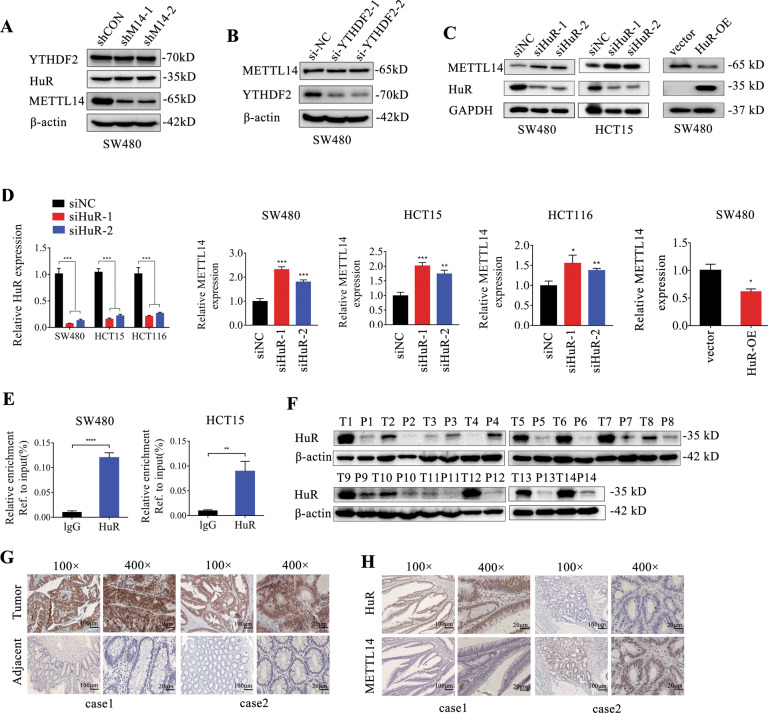
HuR participates in regulating METTL14 transcription control. **A** The protein expression levels of YTHDF2, HuR and METTL14 in METTL14-knockdowned SW480 cells were detected by western blotting. **B**, **C** The effect of YTHDF2 and HuR loss or overexpression on METTL14 protein expression in CRC cells were measured by western blotting, respectively. **D** Relative mRNA expression of METTL14 in CRC cells with HuR knockdown or overexpression were measured by qRT-PCR. **E** ChIP assay was performed in SW480 and HCT15 cells to determine whether HuR could bind to the promoter of METTL14. **F** Immunoblotting analysis of HuR in 14 pairs CRC tissue and para-cancerous tissue. **G** Representative IHC staining images of HuR in tumor and adjacent normal samples. **H** The correlation of HuR and METTL14 expression in CRC tissue. *P < 0.05, **P < 0.01, ***P < 0.001, ****P < 0.0001.

METTL14 loss has no effect on YTHDF2 and HuR expression (Fig. 7A). We wondered whether YTHDF2 and HuR could in turn influence the expression of METTL14. The results showed that knockdown HuR, but not YTHDF2, significantly upregulated METTL14 protein expression in CRC cells (Fig. 7B, C). HuR loss also increased METTL14 mRNA expression (Fig. 7D). In contrast, HuR overexpression deceased METTL14 protein and mRNA expression (Fig. 7C, D). Recently, multiple RNA-binding proteins (RBPs) have been implicated in transcription regulation. Served as transcription factors, they control the expression of target gene by binding gene promoter [28]. As a typical RBP, HuR have been reported to affect promoter activity of some targets [29]. We measured the location of HuR in CRC cells and found that HuR was predominantly located in nucleus (Supplementary Fig. S6A). Next, ChIP assay showed that HuR was capable of directly binding to METTL14 gene promoter (Fig. 7E). As revealed by the TCGA database, the expression of HuR was dramatically upregulated in CRC samples (Supplementary Fig. S6B). Consistently, HuR protein expression was enhanced in representative tumor tissue compared with adjacent para-cancer tissue (Fig. 7F, G). Moreover, there was a close negative correlation between HuR and METTL14 expression in CRC tissue (Fig. 7H). In summary, HuR was proven to inhibit METTL14 expression by directly binding to its promoter.

### TCF4 promotes METTL14 protein expression in CRC cells

**Fig. 8:**
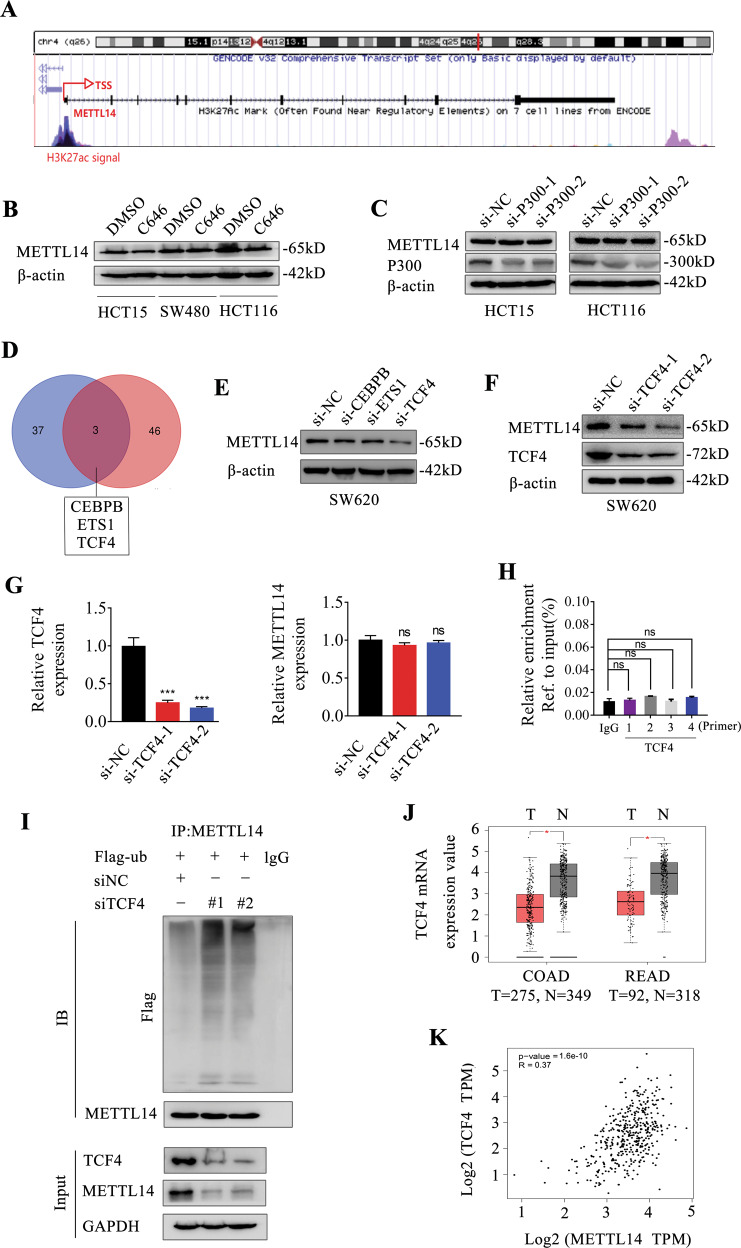
TCF4 regulates METTL14 expression in CRC cells. **A** Data from the UCSC genome bioinformatics site (http://genome.ucsc. edu/) showed high enrichment of H3K27ac in the promoter of METTL14. **B** METTL14 protein expression was determined by western blotting in SW480, HCT116 and HCT15 cells with C646 (20 μm) treatment. **C** METTL14 protein expression was determined by western blot in HCT116 and HCT15 cells with P300 knockdown. **D** Venn diagram shows the overlap of transcription factors of METTL14 predicted by PROMO and ChIPBase, respectively. **E**, **F** METTL14 protein expression was determined by western blotting in SW620 cells with CEBPB, ETS1, TCF4 knockdown, respectively. **G** Relative mRNA expression of METTL14 and TCF4 in SW620 cells with TCF4 knockdown were measured by qRT-PCR. **H** ChIP assay was performed in SW620 cells to determine whether TCF4 could bind to the promoter of METTL14. **I** SW620 cells were transfected with si-NC, siTCF4 or flag-ubiquitin. After METTL14 was immunoprecipitated from equal amounts of lysates, the ubiquitination of METTL14 was examined by western blot. **J** TCF4 expression in the GEPIA date-base. **K** The correlation of TCF4 and METTL14 expression in the GEPIA date-base.

To explore the mechanism of low METTL14 expression in CRC, we first analyzed the modification in the promoter of METTL14 by the UCSC genome bioinformatics site. As shown in Fig. 8A, abundant H3K27 acetylation (H3K27ac) signals were found in the promoter region of METTL14, suggesting that METTL14 might be regulated by chromatin acetylation. H3K27ac is known to be catalyzed by the P300/CBP complex. We then treated CRC cells with C646, a histone acetyltransferase inhibitor targeting P300, and the results showed that METTL14 expression had no obviously change result from C646 treatment (Fig. 8B). Inhibition of P300 with special siRNA also had no effect on METTL14 expression in CRC cells (Fig. 8C), suggesting that low METTL14 expression was regulated by the other mechanism.

We then evaluated the potential transcription factors (TFs) responsible for the regulation of METTL14. To identify TFs that directly regulate METTL14 expression, we analyzed the ENCODE chromatin immunoprecipitation sequencing (ChIP-seq) data in ChIPBase and PROMO. Among the 49 factors identified by ChIPBase and 40 factors identified by PROMO, three TFs including CEBPB, ETS1, and TCF4 were overlapping between the two databases (Fig. 8D). Next, we knockdown these 3 TFs respectively, and found that TCF4 inhibition, but not CEBPB and ETS1, decreased METTL14 expression (Fig. 8E, F). However, TCF4 loss have no obvious effect on METTL14 mRNA expression (Fig. 8G). ChIP assay showed that TCF4 did not bind to METTL14 promoter (Fig. 8H), indicating that TCF4 may influence METTL14 protein stability. Ubiquitination is the most important factor affecting protein stability. To investigate the effect of TCF4 on METTL14 ubiquitination, immunoprecipitation assay was performed. The results showed that TCF4 loss significantly increased METTL14 ubiquitination level, thus promoted its degradation (Fig. 8I). Moreover, GEPIA database showed that TCF4 were low expressed in CRC tissues, and there was a significant positive correlation between TCF4 and METTL14 expression (Fig. 8J, K). In conclusion, TCF4 was proven to regulate METTL14 protein stability in CRC cells.

## Discussions

Besides DNA and histone, RNA can also be chemically modified. Up to now, over 100 types of post-transcriptional modifications have been found in all RNA species [30, 31]. Among these, m6A methylation accounts for more than 80%, and has been identified to exert crucial effect on mRNA splicing, stability, translation and transport [32, 33]. As a reversible and dynamic epigenetic modulation regulating a variety of RNA biological processes, m6A modification is controlled by m6A methyltransferase, demethylases and “reader” proteins [34]. Increasing researches have declared that m6A modification played an important role in the progression of a myriad of tumors [35]. As the pivotal component of m6A methyltransferase complex, METTL14 has been reported to be dysregulated and exerted controversial and diverse biological functions in the progression of various malignancies [6, 20, 36–41]. Herein, we proved that suppression of METTL14 promoted CRC metastasis in vitro and in vivo. METTL14 loss elevated tumor proliferation in vivo but have no obviously effects on proliferation of CRC cells in vitro. We speculated that the complex tumor microenvironment and the longer tumor growth time in vivo amplified the role of METTL14 on proliferation. In CRC, Previous studies have indicated that METTL14 suppressed the tumor progression by regulating miR-375, lncRNA XIST and SOX4, respectively [36, 38, 42]. However, in this study, the novel upstream regulatory molecules and downstream target of METTL14 were identified. We demonstrated that TCF4 promoted METTL14 protein expression and HuR repressed the transcription of METTL14 via interacting with its gene promoter, and thereby prevented the m6A modification of ARRDC4 mRNA. Afterwards, the m6A “reader” protein YTHDF2 directly bound to the m6A site on ARRDC4 mRNA and inhibited its degradation, which ultimately enhanced ARRDC4–mediated metastasis of CRC and resulted in a poor prognosis (Supplementary Fig. S7).

A recent study found that KDM5C-mediated demethylation of H3K4me3 inhibited METTL14 transcription [42]. In present study, H3K27ac signals were also found in the promoter region of METTL14. However, neither C646, a histone acetyltransferase inhibitor, nor P300 knockdown have no effect on METTL14 expression in CRC cells, suggesting that low METTL14 expression was regulated by the other mechanism. HY Weng et.al revealed that transcriptional factor SPI1 was a direct negatively regulator of METTL14 expression in both normal and malignant hematopoietic cells [39]. Though database screening and further verification, TCF4 was identified as the upstream regulatory molecule of METTL14. However, TCF4 regulated METTL14 protein expression level but not mRNA expression. We proved that TCF4 loss increased METTL14 ubiquitination level, thus promoted its degradation. Furthermore, in studying the feedback regulation between METTL14 and RNA-binding protein HuR, we first demonstrated that HuR, functioning as a transcriptional factor, directly bind to the promoter of METTL14 and inhibited its expression in CRC. HuR was overexpressed in CRC and negatively correlated to METTL14 expression, suggesting that it may be a potential therapeutic target for CRC.

Though RNA-sequencing and MeRIP-sequencing, ARRDC4 was elucidated as the pivotal downstream target of METTL14 in CRC for the first time. METTL14 directly bound to ARRDC4 mRNA, promoted gene methylation, and reduced its stability, thereby inhibiting protein expression. As a member of arrestins family, ARRDC4 is vital in glucose metabolism and G-protein-coupled receptor related physiological and pathological processes [43]. ARRDC4 may be a potential target for treating inflammatory triggered by enterovirus 71 [43]. As a key paralogue of thioredoxin interacting protein (TXNIP), ARRDC4 involves in suppressing cancer glycolytic phenotypes under lactic acidosis [24]. Importantly, the expression of TXNIP and ARRDC4 are tightly associated with predicted lactic acidosis pathway activities and correlated with favorable clinical outcomes in human cancers [24]. In this study, we found that ARRDC4 was dramatically upregulated in CRC and negatively correlated with METTL14. Moreover, inhibition of ARRDC4 diminished the metastasis of CRC cells and ZEB1 expression induced by the loss of METTL14, indicating the significance of the METTL14/ARRDC4 axis in suppressing CRC metastasis. However, METTL14 has no obvious effect on ZEB1 targets E-cadherin, N-cadherin expression (date not shown). As a transcriptional factor, ZEB1 can regulate a variety of targets relative to metastasis beyond to EMT markers. For example, ZEB1 controls inflammatory factor IL6, IL8 and IL1β production [44, 45], which can induce cancer metastasis. ZEB1 also can activate signaling pathways associated with tumor metastasis, including PI3K/AKT and WNT5a [46, 47]. It’s worth to investigate the targets of ZEB1 regulating by cooperation of METTL14, YHTDF2, ARRDC4, TCF4 and HuR leads to elevation of invasiveness and migration of colorectal cancer cells in the future study.

M6A “reader” protein can bind to m6A modified motif indirectly or directly to influence RNA function [26]. In this study, RNA pulldown assays demonstrated that YTHDF2 and HuR, but not YTHDC1, IGF2BP1, or YTHDF1/3, could tightly bind to ARRDC4 mRNA. Knockdown of YTHDF2, but not HuR, significantly upregulated ARRDC4 expression, suggesting the central role of YTHDF2. Previous research showed that demethylase ALKBH5 represses pancreatic cancer progression by up-regulating PER1 expression via m6A-YTHDF2-dependent pathway [14]. YTHDF2 has been reported to bind to SOX4 mRNA and induce its degradation [42]. RIP assays further verified that YTHDF2 could bind to ARRDC4 mRNA, suggesting that ARRDC4 was a direct target of YTHDF2 in CRC.

In summary, our findings suggested that METTL14 is decreased in CRC tissues and correlated with CRC patients’ prognosis. Mechanistically, METTL14 hampered CRC metastasis by down-regulating ARRDC4 though an m6A-YTHDF2-dependent manner. The discovery of TCF4 and HuR mediated-METTL14/ARRDC4/ZEB1 axis will provide novel insights into the development of new therapeutic strategies against CRC.

## Supplementary information

SUPPLEMENTAL MATERIAL

Supplementary material

Supplementary material

Related Manuscript File

## Data Availability

The data and materials that support the findings of current study are available from the corresponding authors upon reasonable request.
