# Supplementary material for: TCF4 and HuR mediated-METTL14 suppresses dissemination of colorectal cancer via N6-methyladenosine-dependent silencing of ARRDC4
Source: Cell Death Dis. 2021 Dec 17;13(1):3. doi: 10.1038/s41419-021-04459-0 (PMC8677753; doi:10.1038/s41419-021-04459-0)
Supplement: Supplementary file 1 — SUPPLEMENTAL MATERIAL [file 41419_2021_4459_MOESM1_ESM.doc]

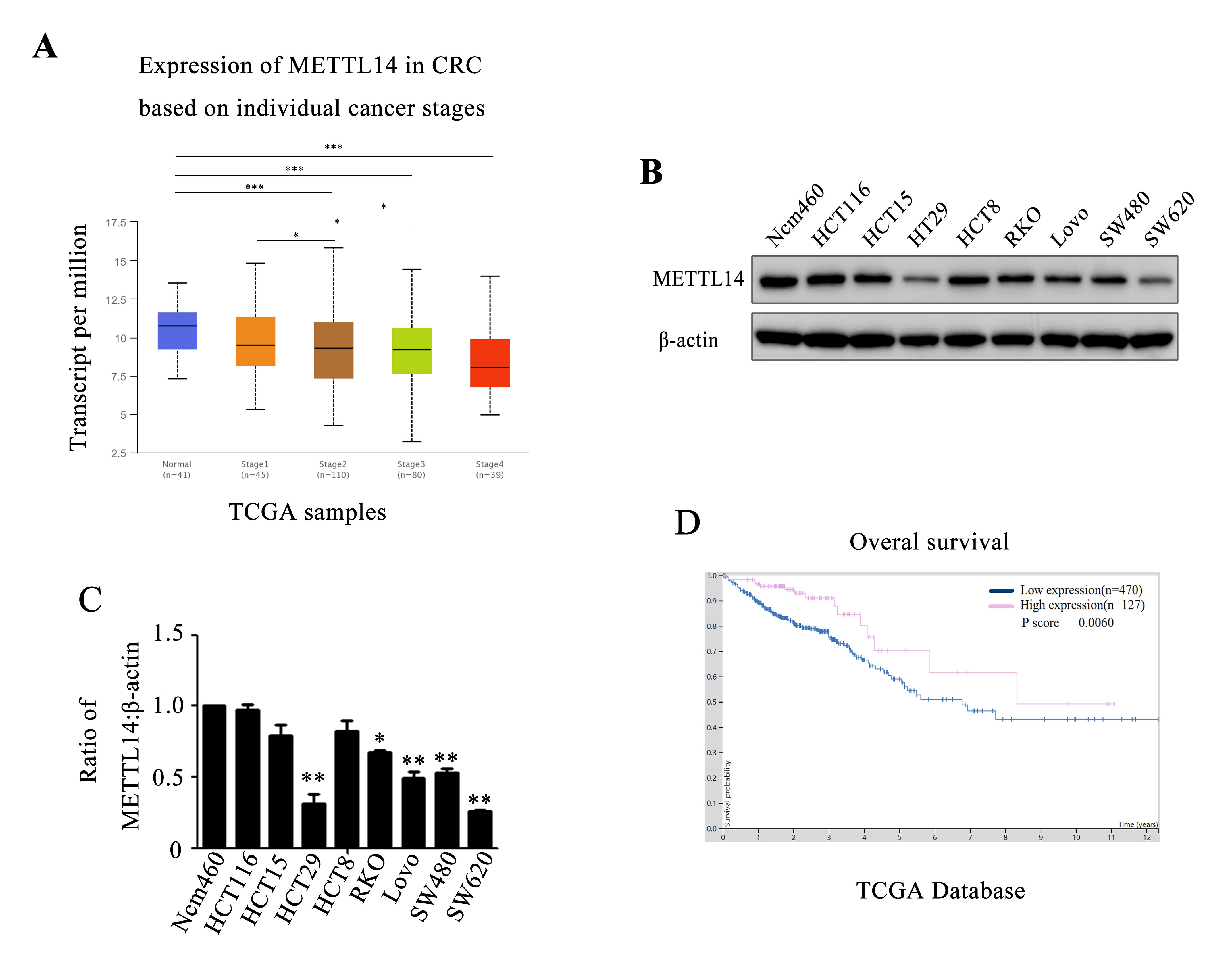


Fig S1. (A) METTL14 expression in CRC based on sample types or tumor stages. (B-C) WB analysis of METTL14 protein expression in CRC cell lines and [intestinal](javascript:;) [epithelium](javascript:;) cells Ncm460. (D) Kaplan-Meier analysis of the correlation between the METTL14 expression and overall survival of CRC patients. *P<0.05, **P<0.01, ***P<0.001.


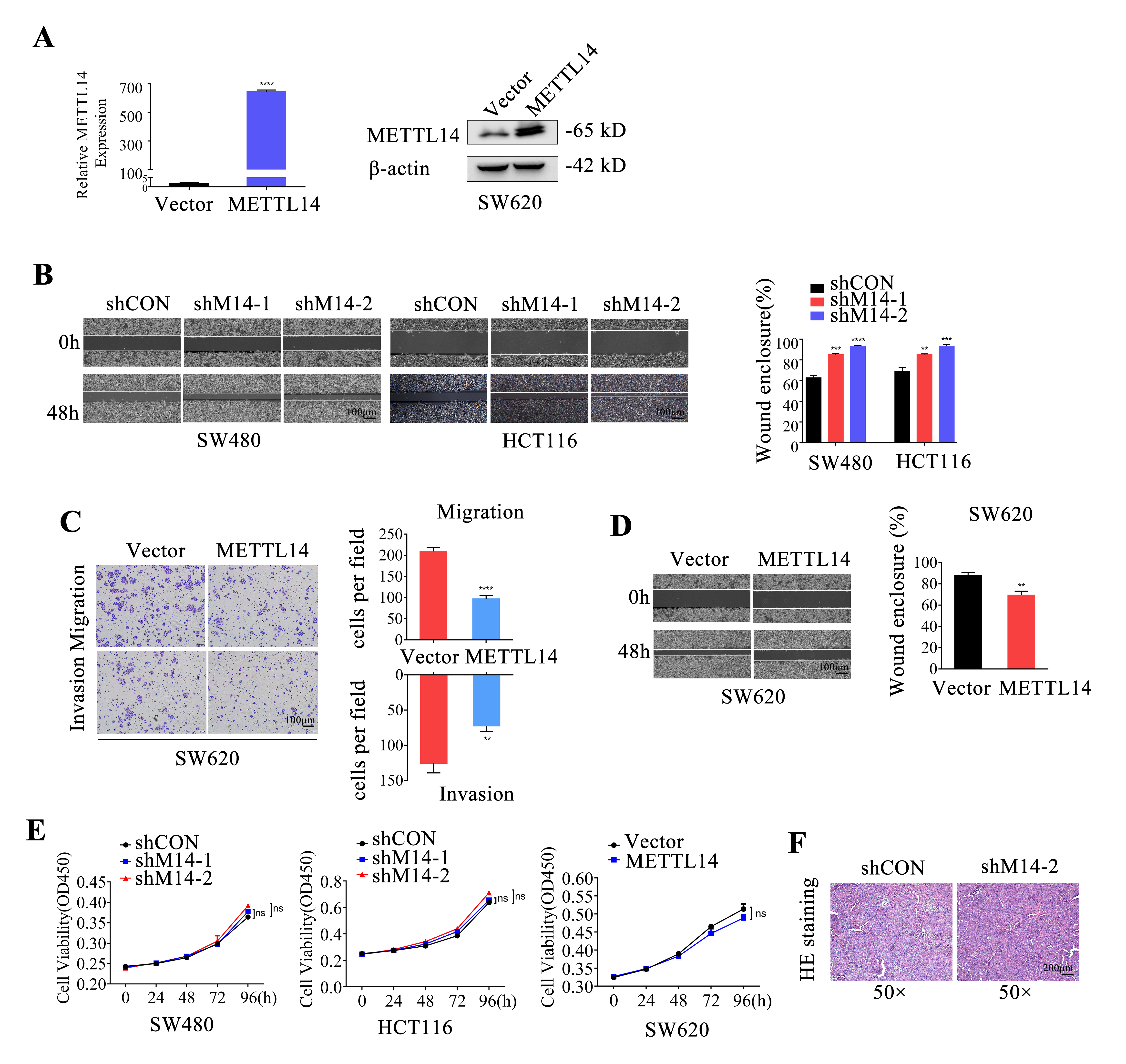


Fig S2. (A) METTL14 overexpression was confirmed in SW620 cell via qRT-PCR and western blot. (B) Representative images and quantification analysis of wound healing assay are managed in METTL14-knockdown SW480 and HCT116 cells. (C) Transwell assays showed the decreased migration and invasion in METTL14-overexpressed SW620 cells. (D) Wound healing assay was conducted in METTL14-overexpressed SW620 cells. (F) [Subcutaneous](javascript:;) [tumor](javascript:;)s collected from different groups were subjected to HE staining (magnification,50×). (E) Proliferation ability of METTL14-silenced SW480 and HCT116 cells, METTL14-overexpressed SW620 cells were determined by CCK8 assay. **P<0.01, ***P<0.001, ****P<0.0001.


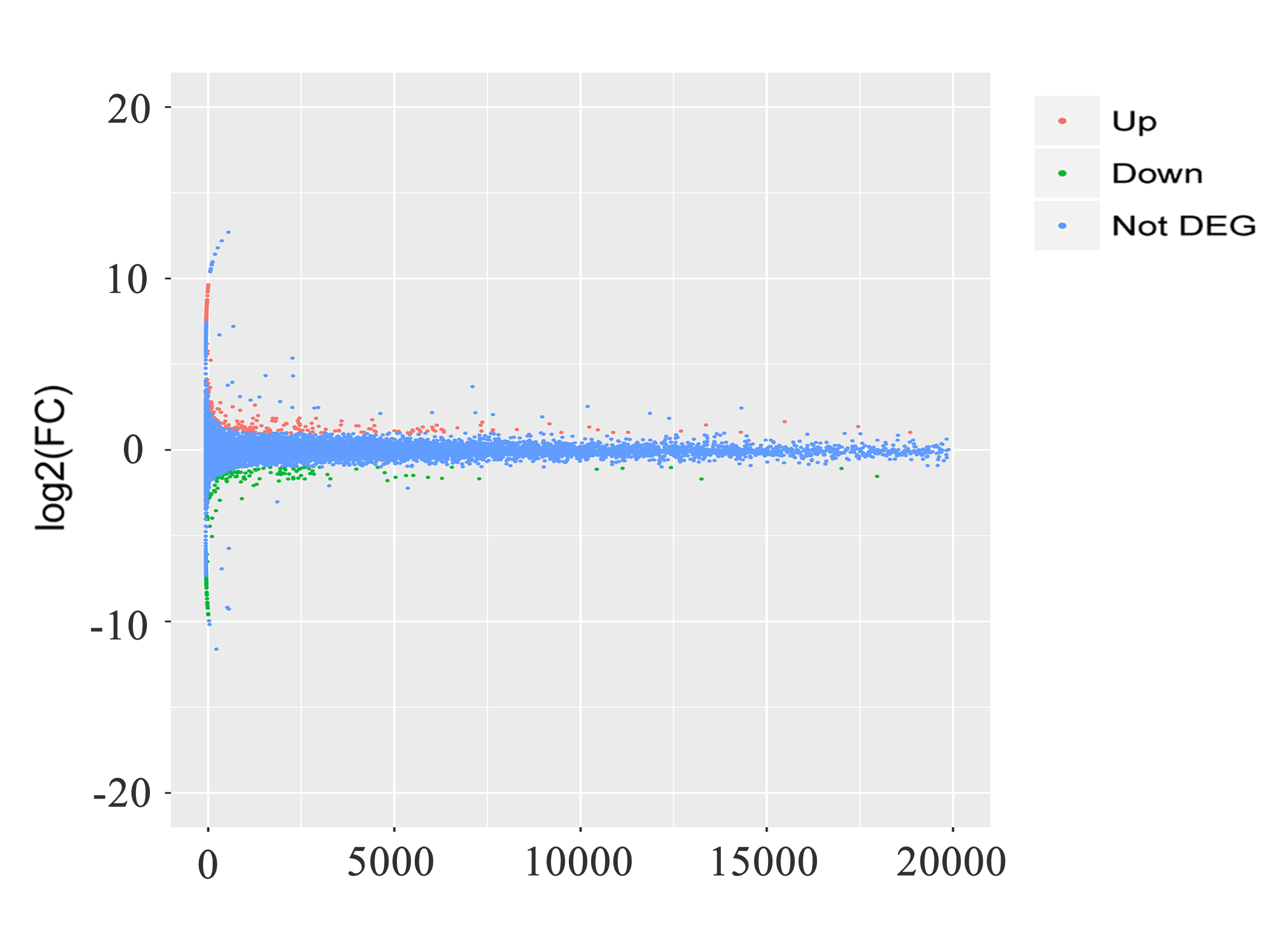


Fig S3. M-A plot showed the upregulated genes (red) and downregulated genes (green) in RNA-sequencing data.


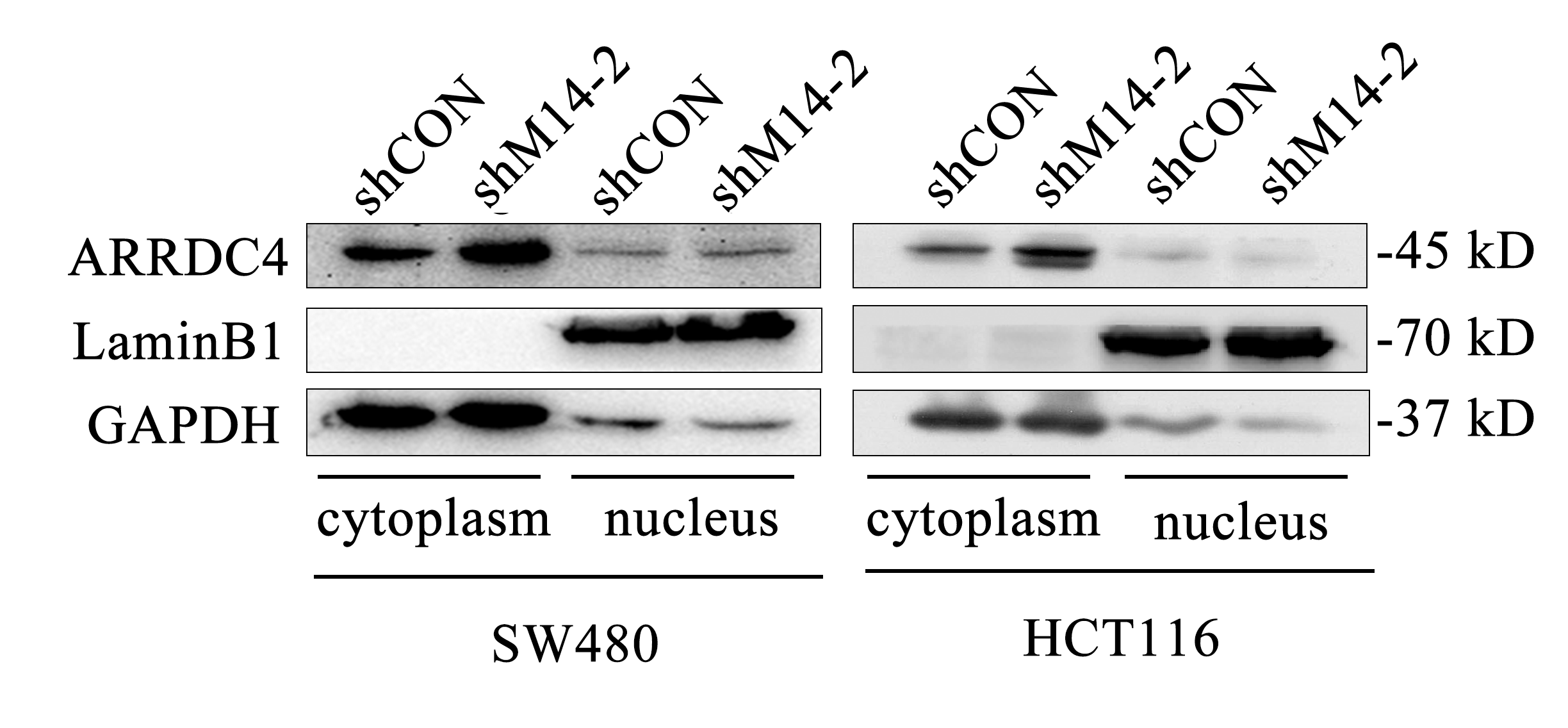


Fig S4. (A) Immunoblotting analysis of ARRDC4 expression in subcellular fractions of SW480 and HCT116 cells stable knockdown and control cells.


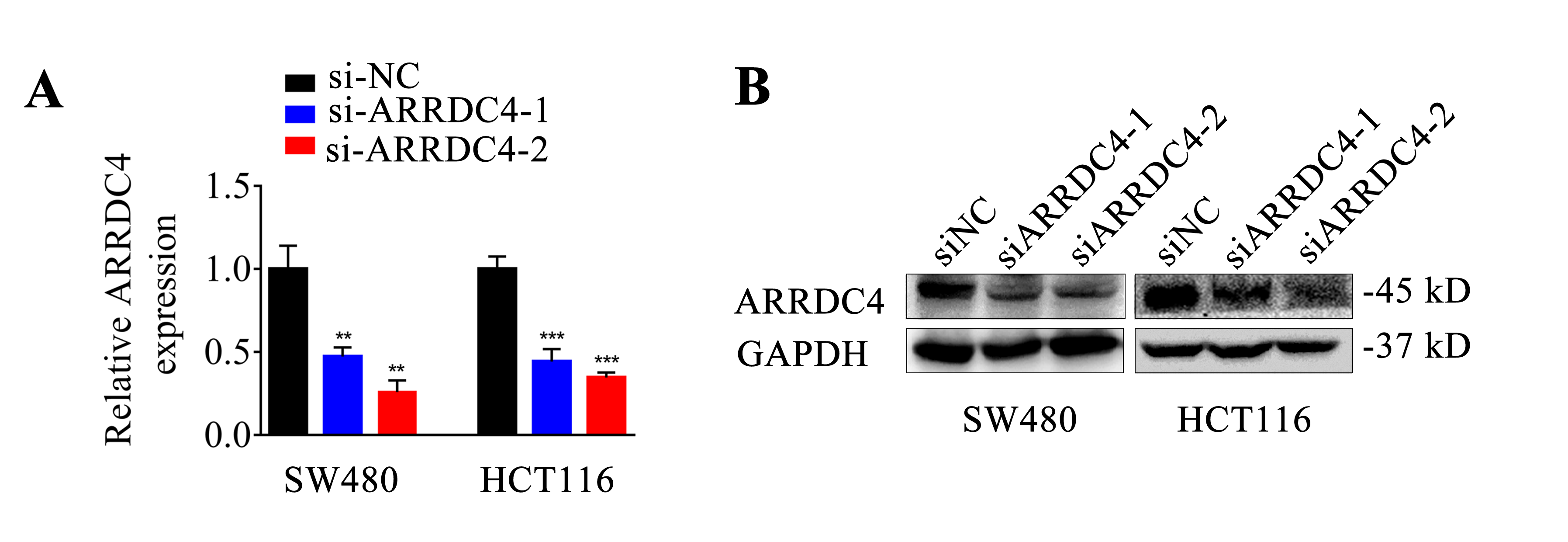


Fig S5. ARRDC4 transient knockdown in SW480 and HCT116 cells were confirmed by qRT-PCR (A) and western blot (B). **P<0.01, ***P<0.001.


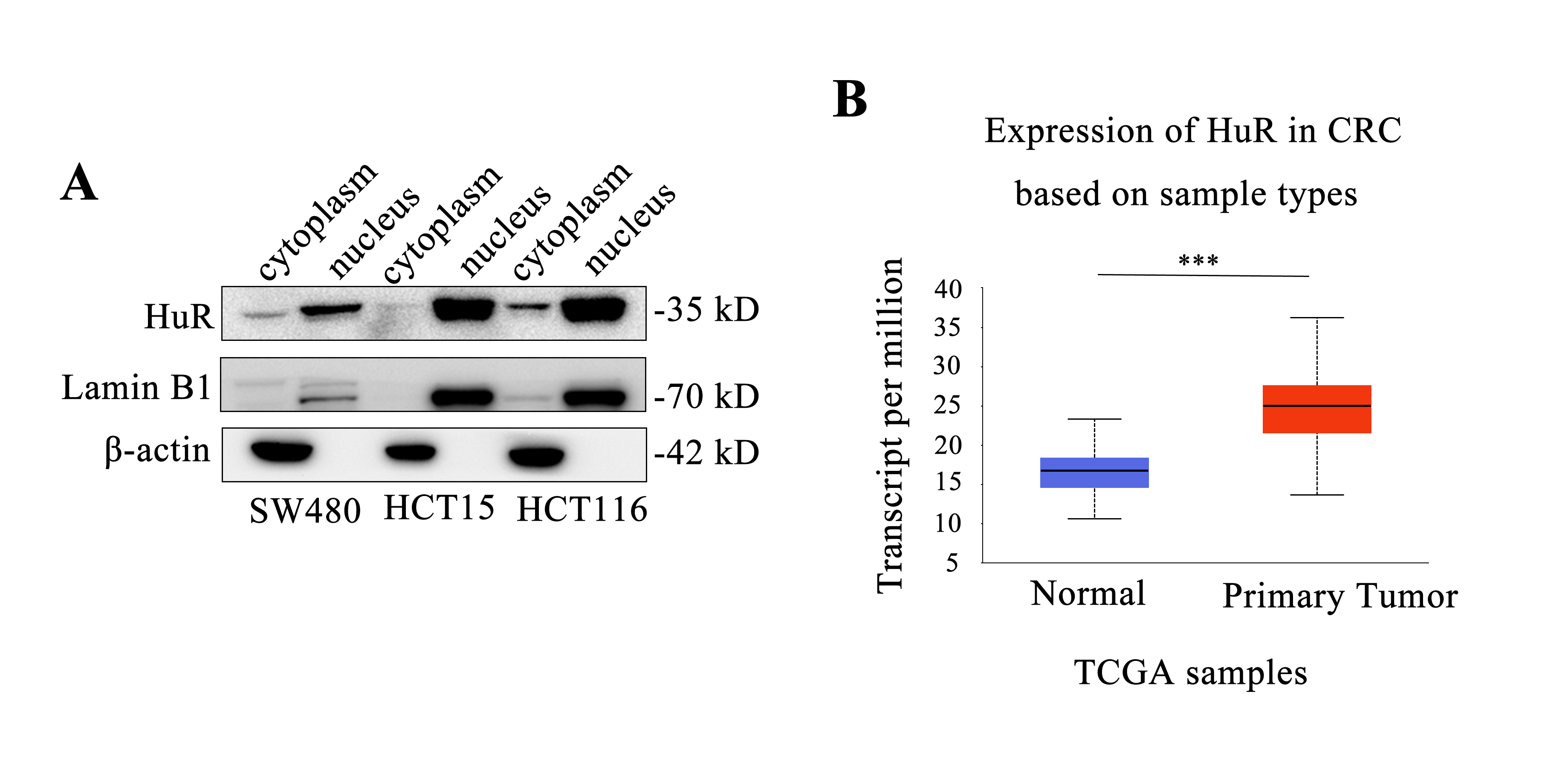


Fig S6. (A) HuR expression in subcellular fractions of SW480, HCT15 and HCT116 cells was verified by western blot. (B) HuR expression in CRC based on sample types in TCGA database. ***P<0.001.


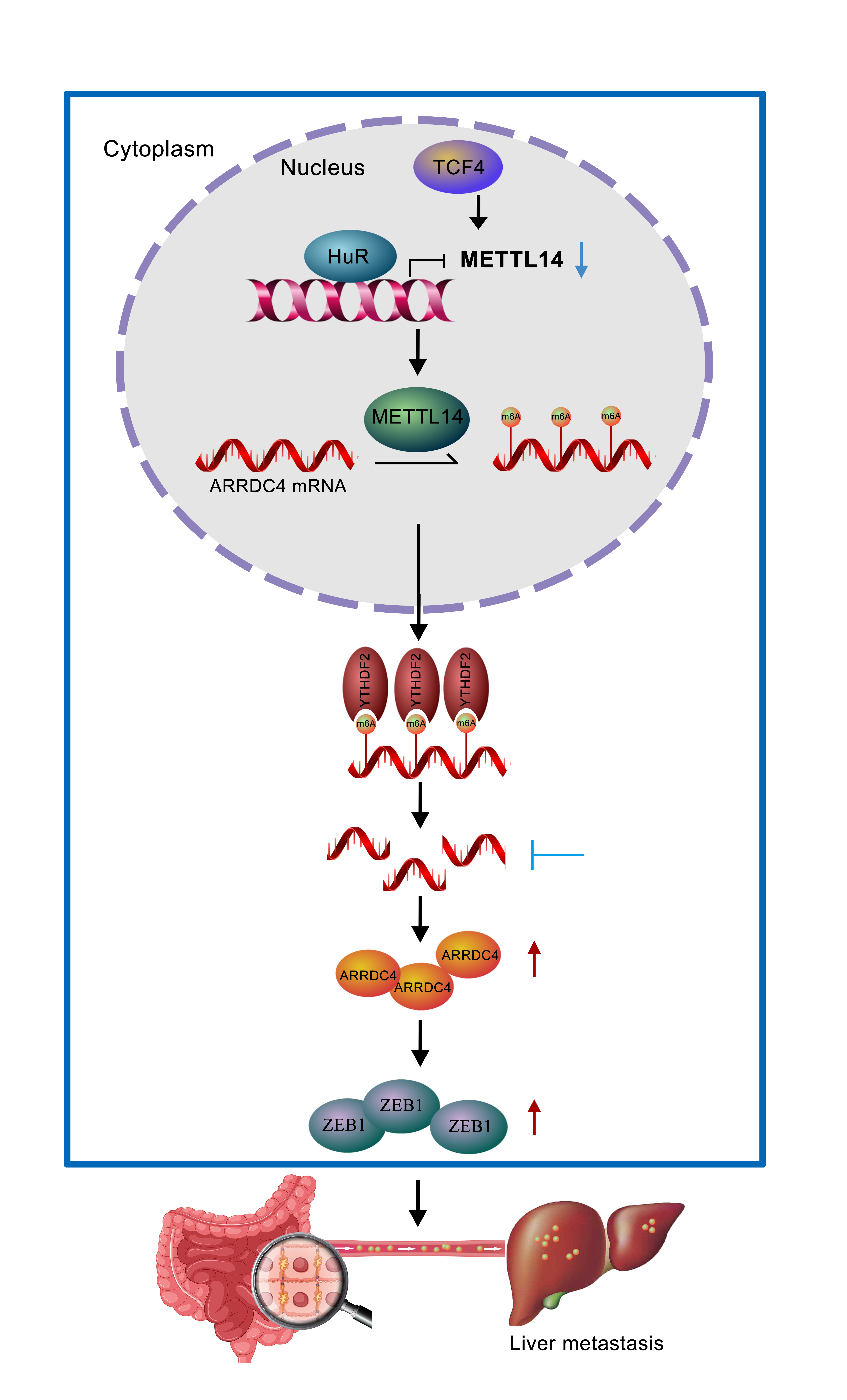


Fig S7. The graphic illustration of METTL14-mediated ARRDC4 expression in promoting metastasis of CRC.

| Primers and RNAi | Sequences（5'-3'） |
| --- | --- |
| METTL14 forward primer | AGAAACTTGCAGGGCTTCCT |
| METTL14 reverse primer | TCTTCTTCATATGGCAAATTTTCTT |
| HuR forward primer | AACTACGTGACCGCGAAGG |
| HuR reverse primer | CGCCCAAACCGAGAGAACA |
| YTHDF2 forward primer | GGCAGCACTGAAGTTGGG |
| YTHDF2 reverse primer | CTATTGGAAGCCACGATGTTA |
| ARRDC4 forward primer | GCAGGAAAGAGTCGCCCG |
| ARRDC4 reverse primer | TAAACTTGTGCGGCAATCCTG |
| TCF4 forward primer | GAAAGCTGCGTGTCTGAAAA |
| TCF4 reverse primer | CATCTGTCCCATGTGATTCG |
| GAPDH forward primer | GGAGCGAGATCCCTCCAAAAT |
| GAPDH reverse primer | GGCTGTTGTCATACTTCTCATGG |
| shMETTL14-1 | GCCGTGTTAAATAGCAAAGAT |
| shMETTL14-2 | GCCGTGGACGAGAAAGAAATA |
| siHuR-1 | GGUUUGGGCGGAUCAUCAA |
| siHuR-2 | GAACGAAUUUGAUCGUCAA |
| siYTHDF2-1 | CCAUGAUUGAUGGACAGUCAGCUU |
| siYTHDF2-2 | GGGUGGAUGGUAAUGGAGUAGGACA |
| siARRDC4-1 | GAGAAGCUAUUCCAAUCUAUU |
| siARRDC4-2 | AAACCAUUAUAUGGAAUUGUA |
| siTCF4-1 | GCUCUGAGAUCAAAUCCGA |
| siTCF4-2 | CGGCACACAUUGUCUCUAA |
| siETS1 | CCGUGCUGACCUCAAUAAGTT |
| siCEBPB | GAAACUUUAGCGAGUCAGA |
|  |  |
| ChIP primers |  |
| For HuR |  |
| METTL14 forward primer | CAGCTCTGTGAGGTAGACACT |
| METTL14 reverse primer | CACAAACTTAAGCCAGATTGCC |
|  |  |
| For TCF4 |  |
| METTL14-1 forward primer | CGCAACAGATCCCTTACTGGG |
| METTL14-1 reverse primer | TCCTCAGTAGAGACTTCCGGC |
| METTL14-2 forward primer | CGTCAGGACTGTCATCTGTGGA |
| METTL14-2 reverse primer | GGGTGGTCTGAGCTAGTCTGA |
| METTL14-3 forward primer | AGTGGGCAGATGTTGTGGC |
| METTL14-3 reverse primer | CACCTTCGTGAGCCTCAGTT |
| METTL14-4 forward primer | ACTTCCGCCACTGTAGGCTT |
| METTL14-4 reverse primer | CATAGGATGGCAGCAAAGGCC |

Table S1. Primers Used in the study.

Table S2. METTL14 expression in clinical and pathological characteristics of colorectal cancer patients.

| **Variable** | **Expression of METTL14 in colorectal cancer** | | | ***P* value** |
| --- | --- | --- | --- | --- |
|  | **High**  **（n=25）** | **Low**  **（n=47）** | **Total**  **(n=72)** |  |
| **Age** |  |  |  | 0.390 |
| <50 | 7 | 9 | 16 |  |
| ≥50 | 18 | 38 | 56 |  |
| **Gender** |  |  |  | 0.714 |
| Male | 16 | 28 | 44 |  |
| Female | 9 | 19 | 28 |  |
| **Tumor size** |  |  |  | 0.888 |
| ≤4 | 10 | 18 | 28 |  |
| >4 | 15 | 29 | 44 |  |
| **Histological grade** |  |  |  | 0.195 |
| Ⅰ-Ⅱ | 23 | 36 | 59 |  |
| Ⅱ-Ⅲ | 2 | 11 | 13 |  |
| **Lymph node** |  |  |  | 0.167 |
| Positive | 8 | 23 | 31 |  |
| Negative | 17 | 24 | 41 |  |
| **Tumor stage** |  |  |  | **0.021 *** |
| T1-T2 | 9 | 6 | 15 |  |
| T3-T4 | 16 | 41 | 57 |  |
